# Validation and Comparison of Accelerometers Worn on the Hip, Thigh, and Wrists for Measuring Physical Activity and Sedentary Behavior

**DOI:** 10.3934/publichealth.2016.2.298

**Published:** 2016-05-20

**Authors:** Alexander H.K. Montoye, James M. Pivarnik, Lanay M. Mudd, Subir Biswas, Karin A. Pfeiffer

**Affiliations:** 1 Clinical Exercise Physiology Program, School of Kinesiology, Ball State University, Muncie, IN, USA; 2 Human Energy Research Laboratory, Department of Kinesiology, Michigan State University, East Lansing, MI, USA; 3 Networked Embedded & Wireless Systems Laboratory, Department of Electrical and Computer Engineering, Michigan State University, East Lansing, MI, USA

**Keywords:** machine learning, artificial neural network, pattern recognition, activity monitor, activity tracker, energy expenditure

## Abstract

**Background:**

Recent evidence suggests that physical activity (PA) and sedentary behavior (SB) exert independent effects on health. Therefore, measurement methods that can accurately assess both constructs are needed.

**Objective:**

To compare the accuracy of accelerometers placed on the hip, thigh, and wrists, coupled with machine learning models, for measurement of PA intensity category (SB, light-intensity PA [LPA], and moderate- to vigorous-intensity PA [MVPA]) and breaks in SB.

**Methods:**

Forty young adults (21 female; age 22.0 ± 4.2 years) participated in a 90-minute semi-structured protocol, performing 13 activities (three sedentary, 10 non-sedentary) for 3–10 minutes each. Participants chose activity order, duration, and intensity. Direct observation (DO) was used as a criterion measure of PA intensity category, and transitions from SB to a non-sedentary activity were breaks in SB. Participants wore four accelerometers (right hip, right thigh, and both wrists), and a machine learning model was created for each accelerometer to predict PA intensity category. Sensitivity and specificity for PA intensity category classification were calculated and compared across accelerometers using repeated measures analysis of variance, and the number of breaks in SB was compared using repeated measures analysis of variance.

**Results:**

Sensitivity and specificity values for the thigh-worn accelerometer were higher than for wrist- or hip-worn accelerometers, > 99% for all PA intensity categories. Sensitivity and specificity for the hip-worn accelerometer were 87–95% and 93–97%. The left wrist-worn accelerometer had sensitivities and specificities of > 97% for SB and LPA and 91–95% for MVPA, whereas the right wrist-worn accelerometer had sensitivities and specificities of 93–99% for SB and LPA but 67–84% for MVPA. The thigh-worn accelerometer had high accuracy for breaks in SB; all other accelerometers overestimated breaks in SB.

**Conclusion:**

Coupled with machine learning modeling, the thigh-worn accelerometer should be considered when objectively assessing PA and SB.

## 1. Introduction

Moderate- to vigorous-intensity physical activity (MVPA; energy expenditure ≥ 3.0 METs), has long been recognized for its beneficial effects on many acute and chronic health indices and is often called “health-enhancing physical activity” [1]. More recently, epidemiologic and laboratory-based studies have uncovered associations between high amounts of sedentary behavior (SB), defined as seated or lying activities requiring low levels of energy expenditure (i.e., ≤ 1.5 METs) [2], and diminished metabolic and cardiovascular health as well as an increased risk of obesity, some cancers, and all-cause mortality [3]–[5]. There is also some evidence that associations of SB and adverse health conditions may be independent of time spent in MVPA [6]. Additionally, the way SB is accrued may influence health, with longer periods of SB being worse than SB broken up periodically by short bouts of non-sedentary activities [6],[7]. While many health-based interventions focus on increasing MVPA, there is also evidence that reducing SB by increasing light-intensity physical activity (LPA; energy expenditure 1.6–2.9 METs) is beneficial to health, even if MVPA is unchanged [8],[9]. However, despite emerging findings of potential SB health risks, current research is insufficient to allow for creation of evidence-based recommendations for SB or how SB and MVPA interact to affect health; contributing to the lack of evidence-based recommendations is the difficulty in accurately measuring time spent in each physical activity (PA) intensity category (SB, LPA, MVPA).

Accelerometry-based activity monitors (accelerometers) are currently used to measure PA intensity category. Traditionally, raw accelerometer data were converted to ‘activity counts,’ which correspond to frequency and magnitude of acceleration. Cut-point thresholds could then be developed to assess SB, LPA, or MVPA from accelerometer data. A range of cut-points are available to assess PA intensity category; perhaps the most commonly used cut-points for adult populations are < 100 counts/minute for SB, 100–1,951 counts/min for LPA, and ≥ 1,952 counts/min for MVPA for the ActiGraph accelerometer [10]–[13]. However, these cut-points were developed in specific populations and during strict, laboratory-based protocols. Other studies validating the ActiGraph have found vastly different cut-points for SB (range 50–250 counts/min) and MVPA (191–2,691counts/min) in adults, depending on the population and type of validation setting [14]–[18]. Regardless of which cut-points are chosen to designate SB, LPA, and MVPA, the cut-point method has several limitations. First, the cut-point approach cannot differentiate standing from sitting/lying, but standing is considered LPA because it elicits different physiologic responses and has different long-term health consequences than sitting/lying [19],[20]. Furthermore, the MVPA threshold for accelerometer counts/min is considerably different for ambulatory activities compared to non-ambulatory activities, rendering any single cut-point inaccurate for assessing MVPA [21]. An accurate measurement tool for SB needs to differentiate between SB and standing or other LPA; the measurement tool must also be able to classify intensity of both ambulatory and non-ambulatory activities for MVPA.

Due to limitations of the cut-point approach to measuring PA intensity categories, researchers have utilized machine learning models to improve accuracy of PA measurement. Studies show improved measurement of energy expenditure, accurate classification of activity type, and correct classification of PA intensity category from a hip-mounted accelerometer [22]–[24]. Despite the common use of hip-mounted accelerometers, there are advantages, such as improved comfort and compliance, of wearing accelerometers on other body locations. Research shows that machine learning modeling has dramatically improved measurement accuracy of accelerometers worn on various body locations, such as the wrist and thigh [23],[25]. Additionally, accelerometers worn on the wrist and thigh have shown strong ability to detect specific activities and have yielded acceptably accurate assessments of energy expenditure and SB [23],[26],[27]. However, these accelerometer placements have not yet been tested for assessment of PA intensity category when coupled with machine learning models. Therefore, the purpose of our study was to develop, validate, and compare the accuracy of hip-, thigh-, and wrist-worn accelerometers, coupled with machine learning models, for measuring 1) total time spent in SB, LPA, and MVPA intensity categories, and 2) breaks in SB in a semi-structured setting.

## 2. Materials and Method

### 2.1. Study participants

Study participants (n = 44, 50% female) were recruited from the surrounding area of East Lansing, MI. Eligible participants were able to perform MVPA safely, did not have orthopedic limitations, were 18–44 years old and could read and speak the English language. Written, voluntary informed consent was obtained from all participants, and this study was approved by the Michigan State University Institutional Review Board.

### 2.2. Equipment

Each participant wore four accelerometers in this study. Two ActiGraph GT3X+ accelerometers (ActiGraph LLC, Pensacola, FL) were worn, one on the midline of the right thigh (adhered with hypoallergenic tape), and one above the right hip at the anterior axillary line (secured on elastic hip belt). Participants also wore two GENEActiv accelerometers (Activinsights Ltd, Kimbolton, Cambridgeshire, UK), one on the dorsal side of each wrist using a manufacturer-supplied watch strap. A sampling frequency of 20 Hz was chosen for the GENEA accelerometers for comparison to another accelerometer tested (not used in this analysis). ActiGraph accelerometers have a minimum sampling frequency of 30 Hz; therefore, 40 Hz was chosen for sampling frequency, which was reintegrated to 20 Hz after downloading data. A portable metabolic analyzer (Oxycon Mobile; CareFusion, San Diego, CA) was worn by participants during the study, but these data were not utilized for the current analysis.

### 2.3. Procedure

Upon arrival at the Human Energy Research Laboratory, each participant's weight and height were then measured (to the nearest 0.1 kg and 0.1 cm, respectively) according to standardized methods [28]. Handedness was determined by asking participants which hand they prefer to use for the majority of everyday activities, and participant age was determined from self-reported date of birth.

After being fitted with the four accelerometers, participants performed 13 activities of different types and intensities that encompassed types of activities that individuals may perform in a free-living environment (Table 1). Participants performed the activities for a total of 90 minutes. They performed each of the activities for between 3–10 minutes each, in the order and exact duration of their choosing. Ambulatory activities (walking and jogging) are common in accelerometer validation literature; we included these but added sedentary, exercise, and lifestyle activities to determine the potential for the four accelerometer placements to measure different PA intensity categories accurately in a semi-structured, simulated free-living setting. The 13 activities were described to each participant prior to the start of the protocol, and some of the less familiar activities (e.g., squats) were demonstrated to ensure understanding. Additionally, the research assistant performing direct observation (DO) updated participants periodically on which activities they still needed to complete.

**Table 1.** Activities performed during the semi-structured protocol.

Activity	PA intensity category
Lying down	SB
Reading	SB
Computer use	SB
Standing	LPA
Laundry	LPA
Sweeping	LPA
Biceps curls (1.3 kg resistance in each hand)	LPA
Walking slow (self-paced)	LPA
Walking fast (self-paced)	MVPA
Jogging (self-paced)	MVPA
Cycling (self-paced)	MVPA
Stair climbing and descending (self-paced)	MVPA
Squats (body weight as resistance)	MVPA

### 2.4. Creation of models to predict PA intensity category

**Figure 1.**
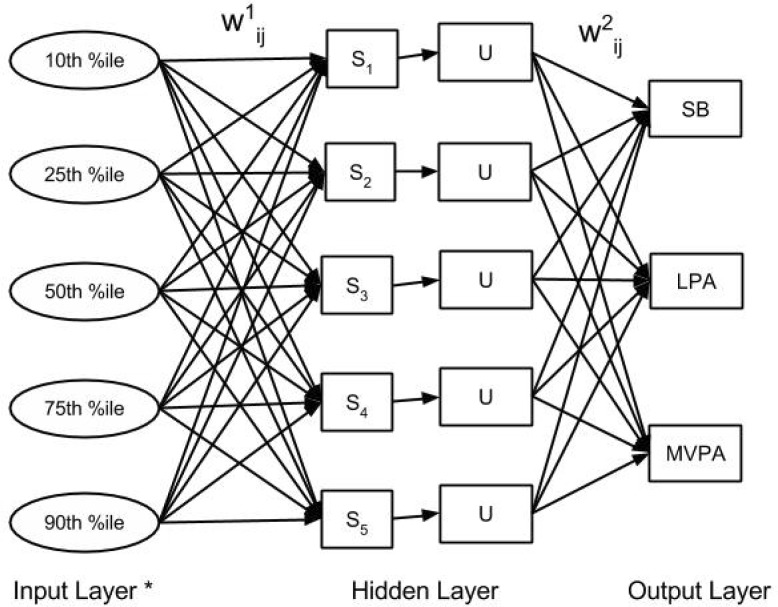
ANN for predicting PA intensity category. *The number of input features was 15 (5 features*3 measurement axes). Abbreviations: 10^th^ % ile: 10^th^ percentile of acceleration signal. 25^th^ % ile: 25^th^ percentile of acceleration signal. 50^th^ % ile: 50^th^ percentile of acceleration signal. 75^th^ % ile: 75^th^ percentile of acceleration signal. 90^th^ % ile: 90^th^ percentile of acceleration signal. S: summation functions of the input layer in the hidden units. U: activation function for the hidden layer. W^1^: the weight vectors for each of the inputs. W^2^: the weight vectors for each of the summations.

From the raw accelerometer data, percentiles (10^th^, 25^th^, 50^th^, 75^th^, and 90^th^) were extracted for each accelerometer axis for each 30-second window of data, and the extracted features were used as inputs for machine learning model development. No filtering of the raw accelerometer data was conducted prior to feature extraction. The 30-second window length was chosen partially to time-match with portable metabolic analyzer data; additionally, 30-second windows were chosen rather than 60-second windows due to non-steady-state nature of the protocol. Artificial neural networks (ANNs), a popularly tested modeling technique for predicting activity type and energy expenditure from accelerometer data, were developed for this study [24],[29],[30]. The ANNs were created to categorically classify all activity into one of three intensity categories: SB, LPA, or MVPA. The decision not to further differentiate MVPA into moderate- or vigorous-intensity PA was made to maximize accuracy of the ANN models, which have lower classification accuracy as more classification categories are added [31]–[33]; additionally, for researchers and practitioners interested in assessing health-enhancing PA rather than specific intensities, further differentiation of higher-intensity activities may not always be necessary. A graphical depiction of the ANNs created in the current study is shown in Figure 1. The ANN function outputs a probability between 0–1 of each PA intensity category. Values closer to one represented a higher likelihood of that intensity category, and the intensity category with the value closest to one was chosen as the predicted output by the ANN. Breaks in SB from the accelerometers were determined as a 30-second window of LPA or MVPA which followed time classified as SB. The ANNs created in this study contained 1 hidden layer and 5 hidden units. Skip-layer connections were not allowed in the ANN.

The ANNs were created and tested using a leave-one-out cross-validation. In this approach, data from all but one participant were used to estimate the weights for each input feature for predicting PA intensity category. Then, the ANN was tested on the data from the participant left out of the training phase by supplying the input features and comparing the predicted PA intensity category from the ANNs to the recorded PA intensity category from DO. The leave-one-out cross-validation is an iterative approach and was repeated with each participant's data used as the testing data once. This process was conducted separately for each accelerometer, resulting in four distinct ANNs. The ANNs developed can be found at the following link: https://drive.google.com/open?id=0B-BgdTzyd2OxazA1UE1zcTFEY1k.

### 2.5. Direct observation

DO served as the criterion measure for time spent in SB, LPA, MVPA, and the number of breaks in SB for the current study. Activities performed were recorded continuously and in real time on a handheld personal digital assistant using the BEST (Observerware, Hobe Sound, FL) observation software. Using this software, activities performed during the visit were coded as 1 of the 13 activity types listed in Table 1. Research assistants were trained to record an activity change as closely as possible to the moment it occurred. After collection, DO data were synchronized with the accelerometer data so that each 30-second window of accelerometer data was matched to the actual activity performed during that window. In most cases, only one activity occurred during a given 30-second window. However, when transitioning between activities, two activities could occur in the same window. If this occurred, the window was automatically recoded as a transition. Additionally, the transition category was used to define all time between activities, such as resting or making an equipment adjustment between activities. Thus, transitions did not represent a specific activity type but instead involved walking, standing, etc. that occurred at the end of one activity and before the next started. All data coded as a transition were included when determining breaks in SB but removed from the DO and accelerometer datasets prior to creation and testing of the ANNs for the determination of time spent in each PA intensity category. From the DO data, activities were classified into one of three intensity categories (SB, LPA, or MVPA) based on MET values published in the Compendium of Physical Activities [34]. For DO, transitions from SB to a non-sedentary activity were summed to obtain total breaks in SB during the protocol. Five research assistants collected DO data during the study. Pilot testing demonstrated inter-researcher reliability of *r* > 0.92 across all research assistants for assessment of total time in each activity type.

### 2.6. Statistical analyses

Sensitivity and specificity were calculated for SB, LPA, and MVPA for each accelerometer and each participant. Sensitivity was calculated by assessing the proportion of instances of a certain intensity category correctly classified as that category by the accelerometer (e.g., the proportion of instances where the participant was performing SB that the thigh-worn accelerometer correctly classified the activity as SB). Specificity was calculated as the proportion of instances where an intensity category was not performed and was correctly classified as not performing that intensity category (e.g., the proportion of time an individual was not performing SB [i.e., was performing LPA or MVPA] that the thigh-worn accelerometer correctly classified the activity as not SB). Repeated measures analysis of variance tests were used to compare sensitivity and specificity among accelerometers. Confusion matrices were created to assess misclassification of PA intensity category, and weighted Kappa statistics (with quadratic weights and equal weighting for relative distance) were calculated and compared among accelerometer placements. Additionally, repeated measures analysis of variance was conducted to compare total time spent in SB, LPA, and MVPA predicted from each accelerometer to time measured by DO. For breaks in SB, criterion-measured breaks were also obtained for each participant using DO. Differences among DO and the four accelerometers were evaluated with repeated measures analysis of variance. If significant differences were revealed in any of the repeated measures analysis of variance tests, post hoc dependent t-tests were conducted with a least significant difference correction. An alpha level of *p* < 0.05 was used to determine statistical significance. We desired 90% power to be able to detect significant differences for an effect size of 0.5 among accelerometers for sensitivity and specificity and for predicted and measured time spent in each PA intensity category and breaks in SB. At an alpha level of *p* = 0.05, 36 subjects were required. Therefore, our sample size of 44 provided sufficient power to address our research questions.

## 3. Results

Of the 44 participants who participated in study, accelerometer malfunction occurred during one participant's visit, resulting in exclusion from the data analysis. Additionally, the portable metabolic analyzer (used to address an aim not part of the current study) malfunctioned in three participants, resulting in premature termination of the protocol and exclusion of their data from the analysis. Demographic characteristics of the 40 participants included in data analysis are displayed in Table 2. Approximately 25% of the sample was classified as overweight (≥ 25.0 kg/m^2^ body mass index), and 90% of the sample reported being right-hand dominant.

**Table 2.** Demographic characteristics of participants in study.

	All (n = 40)	Males (n = 19)	Females (n = 21)
**Age (years)**	22.0 (4.2)	23.7 (5.0)	20.5 (2.7)
**Weight (kg)**	71.9 (16.3)	84.5 (13.1)	60.4 (8.8)
**Height (cm)**	171.0 (10.3)	179.1 (7.7)	163.7 (5.9)
**BMI (kg/m^2^)**	24.3 (3.5)	26.3 (3.4)	22.5 (2.6)
**Left-hand dominant**	n = 4 (10.0%)	n = 4 (21.1%)	n = 0 (0.0%)

Data for age, weight, height, and body mass index (BMI) are displayed as mean (standard deviation).

Data for hand dominance are displayed as total number (% of sample).

Sensitivity and specificity for classification of each PA intensity category are displayed in Table 3. For SB, the thigh-worn accelerometer had significantly higher sensitivity and specificity than the rest of the accelerometers, and the left wrist-worn accelerometer showed higher sensitivity and specificity than the hip- and right wrist-worn accelerometers. For LPA, the thigh-worn accelerometer had significantly higher sensitivity and specificity than the hip- and right-wrist-worn accelerometers, and the left wrist-worn accelerometer had significantly higher sensitivity and specificity than the hip-worn accelerometer. For MVPA, the thigh-worn accelerometer had significantly higher sensitivity and specificity than all other accelerometers, and the left wrist-worn accelerometer had higher sensitivity and specificity than the hip- and right wrist-worn accelerometers; conversely, the right wrist-worn accelerometer had a lower sensitivity and specificity than all other accelerometers.

**Table 3.** Sensitivity and specificity for predictions of SB, LPA and MVPA.

Accelerometer	SB		LPA		MVPA	
	Sensitivity (%)	Specificity (%)	Sensitivity (%)	Specificity (%)	Sensitivity (%)	Specificity (%)
**Hip**	88.3 (13.6)^23^	96.3 (4.2)^23^	94.9 (7.1)^234^	96.9 (3.9)^234^	86.5 (10.5)^234^	92.9 (5.4)^234^
**Thigh**	99.5 (1.9)^134^	99.9 (0.4)^134^	99.6 (1.8)^14^	99.7 (1.3)^14^	99.2 (3.3)^134^	99.5 (1.8)^134^
**Left wrist**	97.5 (4.8)^124^	99.2 (1.4)^124^	99.0 (2.7)^1^	99.3 (1.8)^14^	90.8 (10.3)^124^	95.4 (4.6)^124^
**Right wrist**	93.1 (7.4)^23^	97.6 (2.6)^23^	97.8 (3.6)^12^	98.7 (1.8)^12^	65.7 (19.1)^123^	84.1 (6.5)^123^

Data are displayed as mean (standard deviation).

^1^Indicates significant difference from hip-worn accelerometer. ^2^Indicates significant difference from thigh-worn accelerometer. ^3^Indicates significant difference from left wrist-worn accelerometer. ^4^Indicates significant difference right wrist-worn accelerometer.

**Figure 2.**
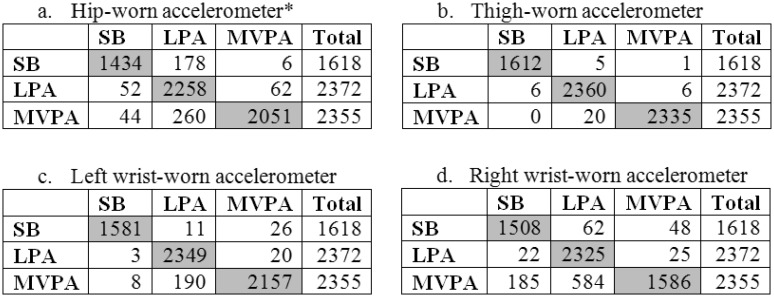
Confusion matrices for prediction of SB, LPA, and MVPA. For a-d, rows are actual PA intensities and columns are predicted PA intensities. Grey boxes represent number of instances where the PA intensity category was correctly predicted.

To further examine PA intensity category prediction, confusion matrices were created for PA intensity classification by each accelerometer, as shown in Figure 2. For the hip-worn accelerometer, most misclassifications were by a single intensity category, with only 6 instances (0.4%) of SB misclassified as MVPA and 44 instances (1.9%) of MVPA misclassified as SB. A similar scenario was present for the thigh-worn accelerometer (1 instance [< 0.1%] of SB misclassified as MVPA and 0 instances of MVPA misclassified as SB) and the left wrist-worn accelerometer (26 instances [1.6%] of SB misclassified as MVPA and 8 instances [0.3%] of MVPA misclassified as SB). For the right wrist-worn accelerometer, there were more instances of misclassification of SB as MVPA (48 instances [3.0%]) and MVPA as SB (185 instances [7.9%]). Additionally, weighted Kappa (K) statistics are shown in Table 4. Significant differences in Kappa statistics were seen among all four accelerometers, with almost perfect PA intensity category classification from the thigh-worn accelerometer (K = 0.99), very good classification accuracy from the left wrist-worn (K = 0.95) and hip-worn (K = 0.90) accelerometers, and good classification accuracy of the right wrist-worn accelerometer (K = 0.78) [35].

**Table 4.** Weighted Kappa statistics for PA intensity category classification by accelerometer placement.

Accelerometer placement	Weighted Kappa
Hip	0.90 (0.89 – 0.91)
Thigh	0.99 (0.99 – 1.00)
Left wrist	0.95 (0.94 – 0.96)
Right wrist	0.78 (0.76 – 0.80)

Data are shown as weighted Kappa (95% confidence interval).

**Figure 3.**
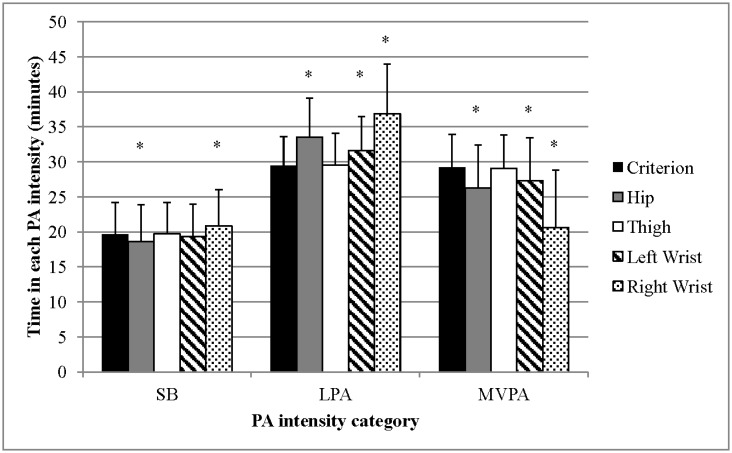
Predicted vs. measured time in each PA intensity category. Error bars represent standard deviation. * Indicates significant differences from the criterion measure (direct observation).

Predictions of total time spent in SB, LPA, and MVPA among accelerometers and the criterion measure (DO) are shown in Figure 3. SB estimated by thigh-worn accelerometer was the same as DO-measured SB for 37 of the 40 study participants, resulting in a mean SB predicted for the entire sample that was not significantly different from DO-measured SB. Additionally, predicted time spent in LPA and MVPA by the thigh-worn accelerometer was not significantly different from DO. Prediction of SB by the left wrist-worn accelerometer was not significantly different than DO, although it overestimated time spent in LPA (2.2 min [∼ 7%], *p* < 0.01) and underestimated time spent in MVPA (1.9 min [7%], *p* < 0.01). Both the hip- and right wrist-worn accelerometers estimates were significantly different from DO for all intensity categories, with the hip-worn accelerometer underestimating SB (1.1 min [6%], *p* = 0.024) and MVPA (3.0 min [10%], *p* < 0.01) but overestimating LPA (4.1 min [15%], *p* < 0.01) and the right wrist-worn accelerometer overestimating SB (1.2 min [6%], *p* < 0.01) and LPA (7.4 min [25%], *p* < 0.01) but underestimating MVPA (8.6 min [30%], *p* < 0.01), compared to DO.

**Figure 4.**
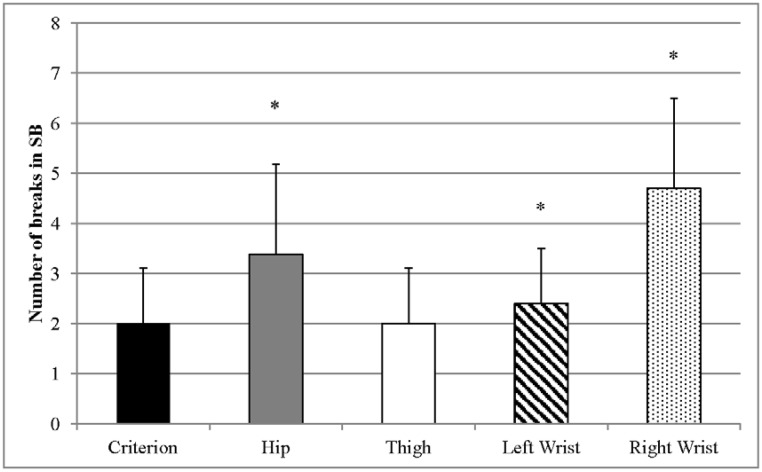
Predicted vs. measured breaks in SB. Error bars represent standard deviation. * Indicates significant differences from the criterion measure (direct observation).

Breaks in SB as predicted by the accelerometers and measured by DO are shown in Figure 4. Breaks in SB predicted by the thigh-worn accelerometer were the same as DO-measured breaks in SB for each of the 40 participants in the study, resulting in the same mean and standard deviation of the data as DO (i.e., was not significantly different from DO). Conversely, breaks in SB were overestimated by the hip-worn accelerometer (mean difference 1.4 breaks [70%], *p* < 0.01), left wrist-worn accelerometer (mean difference 0.4 breaks [20%], *p* < 0.01), and right wrist-worn accelerometer (mean difference 2.7 breaks [135%], *p* < 0.01).

## 4. Discussion

This study's purpose was to test accelerometers worn on the hip, thigh, and wrists (coupled with ANN models) for prediction of time spent in PA intensity categories (SB, LPA, and MVPA) in 30-second windows as well as breaks in SB. While previous research has shown strong utility of ANNs and other machine learning models for assessment of energy expenditure and/or recognizing specific activity types, to our knowledge our study is the first to use machine learning models for several accelerometers specifically to assess time spent in PA intensity categories. The assessment of energy expenditure in terms of Calories or METs has proven difficult, with most current research in this area showing prediction errors (i.e., root mean square error) of > 1.0 MET, which represents a large error when the average daily MET level for adults is < 1.5 METs [23],[36],[37]. While the assessment of PA into three distinct intensity categories (SB, LPA, and MVPA) is more crude than predicting Calories or METs, assessment of time into PA intensity categories is simpler to model and still allows for valuable information regarding individuals' PA levels, adherence to PA guidelines, and associated health implications.

Our study found high accuracy of the thigh-worn accelerometer for predicting time spent in each PA intensity category, as seen by sensitivities and specificities > 99% for correctly classifying each PA intensity category and no differences from the DO in predictions of total time spent in any category; additionally, the thigh-worn accelerometer correctly predicted the number of breaks in SB for all 40 participants in the study, providing evidence that the thigh-worn accelerometer has high utility for detecting temporal changes in PA and SB. Previous research also shows high accuracy of thigh-worn accelerometers for assessing time spent in SB and breaks in SB [14],[15],[27], and our study extends these findings by showing that thigh-worn accelerometers are also capable of accurately assessing time spent in LPA and MVPA, thereby demonstrating the thigh-worn accelerometer's utility to assess time spent in SB and health-enhancing PA (i.e., MVPA). The superior accuracy of the thigh-worn accelerometer supports previous research showing high accuracy of a thigh-worn accelerometer for measurement of energy expenditure and activity type classification [23],[29],[31]. Due to their consistently high accuracy for measuring several different PA constructs and sleep [38], continuous wear (primarily using adhesive tape) for high compliance, and the miniaturization of accelerometer devices, thigh-worn accelerometers have strong potential for assessment of a number of health-related constructs.

Our study also indicated high PA intensity category classification accuracy and prediction of SB for the left wrist-worn accelerometer, achieving sensitivities above 90% and specificities above 95% for all PA intensity categories and no difference from DO for total SB. The left wrist-worn accelerometer did especially well at lower intensity categories, achieving sensitivities and specificities above 97% for SB and LPA. The right wrist-worn accelerometer also had high sensitivities and specificities for SB and LPA (> 93%), but both sensitivity and specificity were significantly lower for MVPA, which was commonly misclassified as LPA. Additionally, the right wrist-worn accelerometer underestimated MVPA but overestimated time spent in SB and LPA and had a more pronounced overestimation of breaks in SB than the left wrist-worn accelerometer. Our findings are supported by those of Esliger et al., who performed the initial validation of the GENEA accelerometer in 2011 and also found higher classification accuracy of PA intensity category using cut-points for a left-wrist accelerometer compared to a right-wrist accelerometer [39]. Given that 90% of our sample reported the left hand being their non-dominant hand, our findings provide evidence that an accelerometer worn on the non-dominant wrist may have better utility for assessing PA intensity category and SB than a dominant wrist-worn accelerometer. One reason for lower accuracy when an accelerometer is worn on the right wrist may be lack of familiarity, since most wristwatches are designed to be worn on the left hand, which could influence movement patterns when performing an activity. Another reason may be the greater variability of movement of the dominant hand during everyday activities. Regardless, these studies provide evidence that studies assessing PA intensity may prefer to use the non-dominant wrist for accelerometer wear. Pavey et al. and Rowlands et al. provide further rationale for using an accelerometer worn on the non-dominant wrist for assessing time spent in SB [26],[40],[41]. These findings also support the choice for wearing accelerometers on the non-dominant wrist in the 2011–2014 NHANES data collection cycle [42].

The hip-worn accelerometer, while providing high specificity across all PA intensity categories, had lower sensitivity for correctly classifying PA intensity category and frequently misclassified SB and MVPA as LPA, resulting in underestimations of time spent in SB and MVPA but an overestimation of time spent in LPA and in breaks in SB. Previous studies by Lyden et al. and Kozey-Keadle et al. demonstrate difficulties in assessing time spent in SB and breaks in SB with hip-worn accelerometers using traditional cut-point methods, and our study adds to these findings by illustrating misclassification of SB as a non-sedentary activity (and vice versa) with machine learning models [14],[15]. Given the similar hip angle and minimal movement present in most types of SB and for non-sedentary activities such as standing, hip-worn monitors appear limited in their capacity for assessing SB. It should be noted that sensitivities and specificities were above 86% and predicted time in each intensity category was within 15% of criterion measure with the hip-worn accelerometer, indicating reasonable accuracy for the hip-worn accelerometer. However, with the high performance of the thigh- and left wrist-worn accelerometers for assessing SB as well as higher-intensity PA, these alternative accelerometer locations, when coupled with machine learning modeling, may be preferable to hip-worn accelerometers for assessment of PA intensity.

There are a number of factors to consider when employing the developed ANNs from our study. First, these ANNs were developed in a semi-structured setting and require cross-validation in a free-living environment. Additionally, most studies use hip-worn accelerometers and cut-points for data analysis, and use of alternate placements and machine learning may affect comparability of PA estimates across studies. Additionally we chose to use 30-second windows due to the non-steady-state nature of the protocol. A 30-second window is shorter than the 60-second window typically used for applying cut-points to accelerometer data in studies assessing PA in adults, and studies evaluating window (or epoch) length show that use of different epoch lengths affects estimations of time spent in different PA intensities [43],[44]. Therefore, the use of alternate accelerometer placements vs. hip-worn accelerometers, machine learning vs. cut-points, and 30-second vs. 60-second windows should be considered when comparing estimates of PA from the developed ANNs to estimates derived from cut-point approaches with hip-worn accelerometers.

This study had several strengths. First, the semi-structured setting allowed for considerable freedom in the manner in which participants performed their activities, thereby improving the generalizability of the ANN models created. The most common accelerometer placement sites (hip, thigh, and wrist) were included in this study, allowing for direct comparison of their utility for assessment of PA intensity category. Moreover, comparison of left and right wrists provided insight into which wrist may be preferable for accelerometer wear. Finally, classification into PA intensity categories, as was performed in this study, allows for reasonably accurate assessment of PA patterns while avoiding the difficulties associated with predicting energy expenditure or activity type using accelerometers.

This study also had several limitations worth considering. The study sample consisted mainly of younger adults, and the ANN models developed in this study need further validation before use in an older or more diverse population. Additionally, while we consider the semi-structured setting a study strength, the study included a limited set of activities, and the ANN models developed in this study may not correctly classify intensity of other activities, such as sports, which were not evaluated in this study.

DO was used as a criterion measure of PA intensity category, which does not directly measure the energy cost of an activity but, rather, classifies it based on the Compendium of Physical Activities [34]. Our choice for using DO instead of directly-measured energy expenditure (e.g., via a metabolic analyzer) to characterize PA intensity category was predicated on the non-steady-state nature of the study protocol. Periods of SB following higher-intensity PA may have a falsely elevated energy cost (and vice versa for higher-intensity PA following SB) as the body's metabolic processes lag behind the current energy cost of the activity, and this would be problematic for assessing PA intensity category using a metabolic analyzer. To illustrate this point, a previous study by our research group using the same participants and activity protocol found that 29.5% of the time participants spent lying, reading, and using the computer (sedentary activities) elicited an energy expenditure > 1.5 METs and 3.3% of the time elicited an energy expenditure ≥ 3.0 METs (measured with a metabolic analyzer), which would incorrectly characterize the activity intensity as LPA and moderate-intensity PA, respectively. Similarly, 2.6% and 3.2% of the time participants spent jogging and using the stairs (MVPA) elicited an energy expenditure < 3.0 METs, which would incorrectly characterize the activity intensity as LPA [23]. Therefore, we feel that the use of DO was preferable over measured energy expenditure for characterizing PA intensity in the current study.

Another study limitation is that our ANN models were not designed to differentiate between moderate-intensity PA (3.0–5.9 METs) and vigorous-intensity PA (≥ 6.0 METs), instead grouping these into a single MVPA category. While our main goal was to develop models capable of differentiating SB, LPA, and health-enhancing PA (MVPA), further differentiation into moderate and vigorous intensity categories would allow for more specific assessment of meeting PA recommendations. Finally, two different brands of accelerometers were used in the current analysis, making it possible that differences seen between the wrists (GENEA accelerometers) and hip and thigh (ActiGraph accelerometers) were due to accelerometer brand. A study by John et al. found that time domain features (similar to those used in the current study) were not interchangeable between monitor brands, providing evidence that ANNs from the wrist-worn GENEA accelerometer would have lower accuracy if used with wrist-worn ActiGraph accelerometers. However, John et al. also found similar PA measurement accuracy when using ANNs developed specifically for each two accelerometer brand [45]. Therefore, it is unlikely that accelerometer brand affected measurement accuracy.

## 5. Conclusion

An accelerometer worn on the right thigh, coupled with an ANN model, achieved high accuracy for classification of three distinct PA intensity categories (SB, LPA, and MVPA) as well as breaks in SB in a semi-structured setting. An accelerometer worn on the left wrist also had high accuracy for assessment of SB but had some misclassification of LPA and MVPA, whereas accelerometers worn on the right wrist and hip had the lowest accuracy for assessment of all PA intensity categories and for measuring breaks in SB. These findings support the use of a thigh-worn accelerometer for assessment of time spent in different PA intensity categories. Alternately, for researchers using wrist-worn accelerometers to assess PA, wear on the non-dominant wrist is likely to allow for higher measurement accuracy than wear on the dominant wrist. Further research should cross-validate these ANN models in a free-living setting to confirm findings from this study.

## References

[1] U.S. Department of Health and Human Services Physical Activity Guidelines Advisory Committee: 2008. Physical Activity Guidelines for Americans.

[2] Sedentary Behavior Research Network (2012). Letter to the Editor: Standardized use of the terms “sedentary” and “sedentary behaviours”. Appl Physiol Nutr Metab.

[3] Hamilton MT, Hamilton DG, Zderic TW (2007). Role of low energy expenditure and sitting in obesity, metabolic syndrome, type 2 diabetes, and cardiovascular disease. Diabetes.

[4] Hu FB, Li TY, Colditz GA (2003). Television watching and other sedentary behaviors in relation to risk of obesity and type 2 diabetes mellitus in women. JAMA.

[5] Katzmarzyk PT, Church TS, Craig CL (2009). Sitting time and mortality from all causes, cardiovascular disease, and cancer. Med Sci Sports Exerc.

[6] Owen N, Healy GN, Matthews CE (2010). Too much sitting: the population health science of sedentary behavior. Ex Sport Sci Rev.

[7] Healy GN, Dunstan DW, Salmon J (2008). Breaks in sedentary time: beneficial associations with metabolic risk. Diabetes Care.

[8] Matthews CE, Moore SC, Sampson J (2015). Mortality Benefits for Replacing Sitting Time with Different Physical Activities. Med Sci Sports Exerc.

[9] Stamatakis E, Rogers K, Ding D (2015). All-cause mortality effects of replacing sedentary time with physical activity and sleeping using an isotemporal substitution model: a prospective study of 201,129 mid-aged and older adults. Int J Behav Nutr Phys Act.

[10] Treuth MS, Schmitz K, Catellier DJ (2004). Defining accelerometer thresholds for activity intensities in adolescent girls. Med Sci Sports Exerc.

[11] Freedson PS, Melanson E, Sirard J (1998). Calibration of the Computer Science and Applications, Inc. accelerometer. Med Sci Sports Exerc.

[12] Pedisic Z, Bauman A (2015). Accelerometer-based measures in physical activity surveillance: current practices and issues. Br J Sports Med.

[13] Healy GN, Clark BK, Winkler EA (2011). Measurement of adults' sedentary time in population-based studies. Am J Prev Med.

[14] Kozey-Keadle S, Libertine A, Lyden K (2011). Validation of wearable monitors for assessing sedentary behavior. Med Sci Sports Exerc.

[15] Lyden K, Kozey Keadle SL, Staudenmayer JW (2012). Validity of two wearable monitors to estimate breaks from sedentary time. Med Sci Sports Exerc.

[16] Hendelman D, Miller K, Baggett C (2000). Validity of accelerometry for the assessment of moderate intensity physical activity in the field. Med Sci Sports Exerc.

[17] Strath SJ, Bassett DR Jr, Swartz AM (2003). Comparison of MTI accelerometer cut-points for predicting time spent in physical activity. Int J Sports Med.

[18] Swartz AM, Strath SJ, Bassett DR (2000). Estimation of energy expenditure using CSA accelerometers at hip and wrist sites. Med Sci Sports Exerc.

[19] Bey L, Hamilton MT (2003). Suppression of skeletal muscle lipoprotein lipase activity during physical inactivity: a molecular reason to maintain daily low-intensity activity. J Physiol.

[20] Katzmarzyk PT (2014). Standing and mortality in a prospective cohort of Canadian adults. Med Sci Sports Exerc.

[21] Lyden K, Kozey SL, Staudenmeyer JW (2011). A comprehensive evaluation of commonly used accelerometer energy expenditure and MET prediction equations. Eur J Appl Physiol.

[22] Staudenmayer J, Pober D, Crouter S (2009). An artificial neural network to estimate physical activity energy expenditure and identify physical activity type from an accelerometer. J Apple Physiol.

[23] Montoye AH, Mudd LM, Biswas S (2015). Energy Expenditure Prediction Using Raw Accelerometer Data in Simulated Free Living. Med Sci Sports Exerc.

[24] Lyden K, Keadle SK, Staudenmayer J (2013). A Method to Estimate Free-Living Active and Sedentary Behavior from an Accelerometer. Med Sci Sports Exerc.

[25] Preece SJ, Goulermas JY, Kenney LP (2009). Activity identification using body-mounted sensors--a review of classification techniques. Physiol Meas.

[26] Rowlands AV, Olds TS, Hillsdon M (2014). Assessing sedentary behavior with the GENEActiv: introducing the sedentary sphere. Med Sci Sports Exerc.

[27] Steeves JA, Bowles HR, McClain JJ (2015). Ability of thigh-worn ActiGraph and activPAL monitors to classify posture and motion. Med Sci Sports Exerc.

[28] Malina R (1995). Anthropometry. Physiological assessment of human fitness.

[29] Dong B, Montoye A, Moore R (2013). Energy-aware activity classification using wearable sensor networks.

[30] Montoye A, Dong B, Biswas S (2014). Use of a wireless network of accelerometers for improved measurement of human energy expenditure. Electronics.

[31] Skotte J, Korshoj M, Kristiansen J (2014). Detection of physical activity types using triaxial accelerometers. J Phys Act Health.

[32] Cleland I, Kikhia B, Nugent C (2013). Optimal placement of accelerometers for the detection of everyday activities. Sensors.

[33] Montoye AHK, Mudd LM, Pivarnik JM (2016). Comparison of activity type classification accuracy from accelerometers worn on the hip, wrists, and thigh in young, apparently healthy adults. Meas Phys Ed Exerc Sci.

[34] Ainsworth BE, Haskell WL, Herrmann SD (2011). Compendium of Physical Activities: a second update of codes and MET values. Med Sci Sports Exerc.

[35] Altman D (1991). Practical Statistics for Medical Research.

[36] Di Pietro L, Dziura J, Blair SN (2004). Estimated change in physical activity level (PAL) and prediction of 5-year weight change in men: the Aerobics Center Longitudinal Study. Int J Obes Relat Metab Disord.

[37] Staudenmayer J, He S, Hickey A (2015). Methods to estimate aspects of physical activity and sedentary behavior from high-frequency wrist accelerometer measurements. J Appl Physiol.

[38] Edwardson C, Winkler E, Bodicoat D (2016). Considerations when using the activPAL monitor in field based research with adult populations. J Sport Health Sci.

[39] Esliger DW, Rowlands AV, Hurst TL (2011). Validation of the GENEA Accelerometer. Med Sci Sports Exerc.

[40] Rowlands AV, Yates T, Olds TS (2016). Sedentary Sphere: Wrist-Worn Accelerometer-Brand Independent Posture Classification. Med Sci Sports Exerc.

[41] Pavey TG, Gomersall SR, Clark BK (2016). The validity of the GENEActiv wrist-worn accelerometer for measuring adult sedentary time in free living. J Sci Med Sport.

[42] Troiano RP, McClain JJ, Brychta RJ (2014). Evolution of accelerometer methods for physical activity research. Br J Sports Med.

[43] Orme M, Wijndaele K, Sharp SJ (2014). Combined influence of epoch length, cut-point and bout duration on accelerometry-derived physical activity. Int J Behav Nutr Phys Act.

[44] Ayabe M, Kumahara H, Morimura K (2013). Epoch length and the physical activity bout analysis: an accelerometry research issue. BMC Res Notes.

[45] John D, Sasaki J, Staudenmayer J (2013). Comparison of raw acceleration from the GENEA and ActiGraph GT3X+ activity monitors. Sensors.

